# Supplementary material for: Direct in situ photolithography of perovskite quantum dots based on photocatalysis of lead bromide complexes
Source: Nat Commun. 2022 Nov 7;13:6713. doi: 10.1038/s41467-022-34453-9 (PMC9640639; doi:10.1038/s41467-022-34453-9)
Supplement: Supplementary file 1 — Supplementary Information [file 41467_2022_34453_MOESM1_ESM.pdf]

Supplementary Information

Direct in-situ photolithography of perovskite quantum dots based on photocatalysis of lead bromide complexes

Pingping Zhang,[1] Gaoling Yang,[2*] Fei Li,[3] Jianbing Shi,[1] Haizheng Zhong[1]

1. MIIT Key Laboratory for Low Dimensional Quantum Structure and Devices, School of Materials Science and Engineering, Beijing Institute of Technology, Beijing 100081, China
2. MIIT Key Laboratory for Low Dimensional Quantum Structure and Devices, School of Optics and Photonics, Beijing Institute of Technology, Beijing 100081, China
3. QD LAB, Hefei Innovation Research Institute of Beihang University, Hefei, Anhui, 230001, China

KEYWORDS: Perovskite quantum dots, photolithography, lead bromide complexes, in situ fabrication, patterning

* Corresponding Author

Gaoling Yang: glyang@bit.edu.cn

This file includes 28 pages, 23 figures, 8 tables, and 2 discussions:

Supplementary Table 1 Curing results of different monomers in inks.

Entry	Monomers	Curing results under 405 nm light (3 W)
1	1 TTMP + 1 TAIC	Cured/30 s
2	2 TTMP	Uncured/120 s
3	2 TAIC	Uncured/120 s

Note: a) all entries above were conducted by adding monomers in the perovskite precursor solution with the same concentration. The perovskite precursor solution was prepared by mixing MABr and $PbBr_2$ at a mole ratio of 2:1 in the mixture of DMF and DMSO (v/v = 4:1); b) the numbers before monomer abbreviations are the ratio of monomers; (c) UV LED of 405 nm (3 W) was used to irradiate samples from the same distance in the meantime.

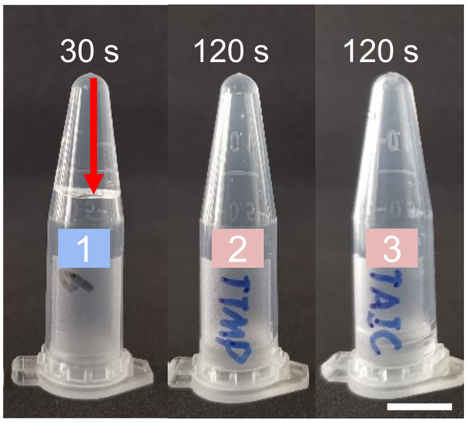

Supplementary Fig. 1 Curing results of different monomers in inks. Photograph of entries 1, 2, 3 in Supplementary Table 1 after 405 nm UV irradiating for 30 s, 120 s, 120 s respectively. The scale bar is 1 cm. Note that the cured products are marked by a red arrow in the figure.

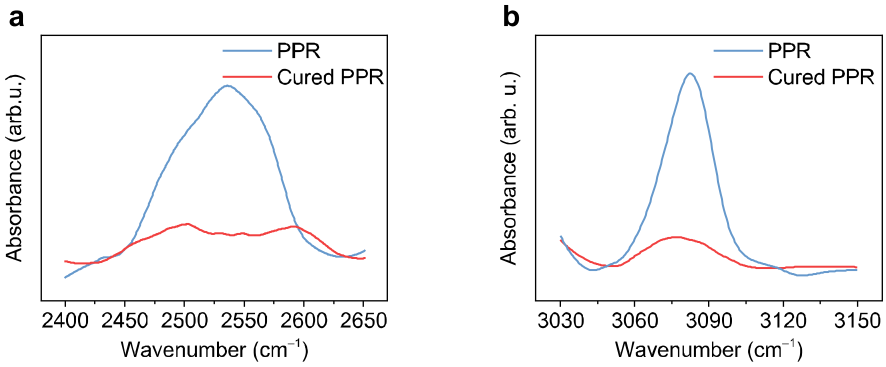

Supplementary Fig. 2 FT-IR spectra of the PPR and cured PPR for thiol-ene polymerization analysis. a FT-IR spectra between 2670 and 2380 cm^{-1}, where the peak at 2527 cm^{-1} represents the vibration of the thiol group. **b** FT-IR spectra between 3160 and 3020 cm^{-1} where the peak at 3080 cm^{-1} represents the vibration of ethenyl group. These measurements were performed by placing 50 μL of sample on the ATR crystal plate. The UV LED (365 nm, 5 W) radiated onto the sample from a distance of 1 cm centered at the ATR crystal plate. Both peaks decayed after exposing PPR for 100 s. Source data are provided as a Source Data file.

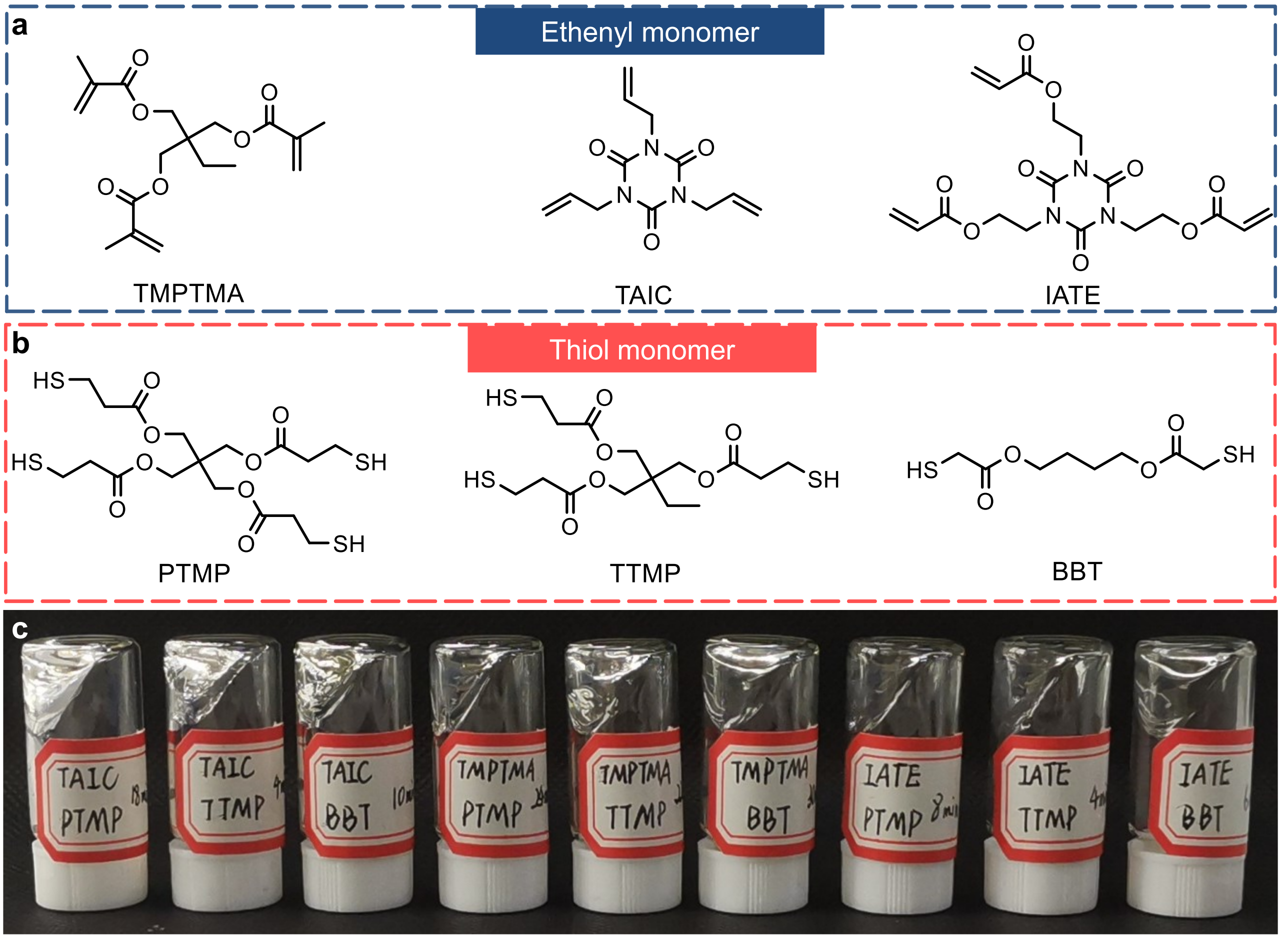

Supplementary Fig. 3 Chemical structures and curing results. a Ethenyl monomers (TMPTMA: Trimethylolpropane trimethacrylate, TAIC: Triallyl isocyanurate; IATE: isocyanuric acid tris(2-acryloyloxyethyl) ester). **b** Thiol monomers (PTMP: Pentaerythritol tetra(3-mercaptoproionate); TTMP: Trimethylolpropane tris(3-mercaptopropionate); BBT: 1,4-Butanediol bis(thioglycolate)). **c** Photograph of curing results of different combinations within 30 min UV light irradiation, each combination was prepared by mixing certain volume of 0.15 M perovskite precursor solution and monomers with identical amount of thiol group and ethenyl group, then irradiated by a 365 nm UV light from the same distance.

Supplementary Table 2 Curing results of different combinations of given ethenyl monomers and thiol monomers in Supplementary Fig. 3c.

	TMPTMA	TAIC	IATE
PTMP	y	y	y
TTMP	y	y	y
BBT	y	y	y

Note: a) “y” represents the combination can be polymerized with existence of perovskite precursor solution.

Supplementary Table 3 Curing results of different additives in inks.

Entry	Monomers	Additives	Cure results under 365 nm light (5 W)
4	1 TTMP + 1 TAIC	None	Cured/60 s
5	1 TTMP + 1 TAIC	HCl (5 v%)	Cured/19 s
6	1 TTMP + 1 TAIC	DMPO (0.5 v%)	Uncured/120 s

Note: a) all entries above were conducted by blending monomers and additives in the perovskite precursor solution with the same concentration. The perovskite precursor solution was prepared by mixing MABr and $PbBr_2$ at a mole ratio of 2:1 in the mixture of DMF and DMSO (v/v = 4:1); b) the numbers before monomer abbreviations are the ratio of monomers; c) UV LED of 365 nm (5 W) was used to irradiate 300 μL liquid of each entry from the same distance.

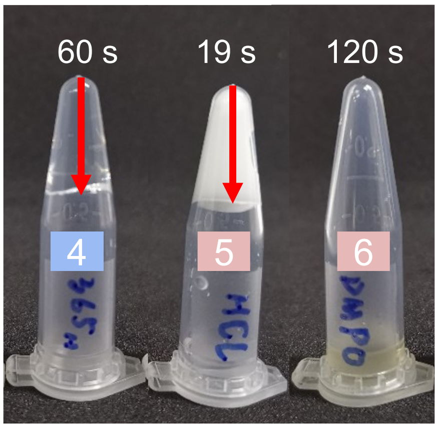

Supplementary Fig. 4 Curing results of different additives in inks. Photograph of entries 4, 5, 6 in Supplementary Table 3 after 365 nm UV LED irradiating for 60 s, 19 s,120 s. Note that cured products are marked by a red arrow in the figures.

Supplementary Table 4 Curing results of different reagent salts in precursor solutions.

Entry	Reagent salts	UV exposure time (~10 mW cm^{-2})			
		4 min	6 min	20 min	30 min
7	2MABr + 1 $PbBr_2$	Cured ~80%	Cured ~100%	Cured	Cured
8	2 $PbBr_2$	Uncured	Uncured	Cured ~40%	Cured ~75%
9	4 MABr	Uncured	Uncured	Uncured	Uncured

Note: a) all entries above were conducted by blending reagent salts and monomers in the DMSO solvent, where the monomers are equal mole TAIC and TTMP with certain concentrations; b) the numbers before reagent salts are assigned to the ratios; c) 365 nm UV light (~10 mW cm^{-2}) was used to irradiate 200 μL liquid of each entry; d) The conversion rate shown here was determined by measuring the volumes of liquid residues and comparing with the original volume of inks.

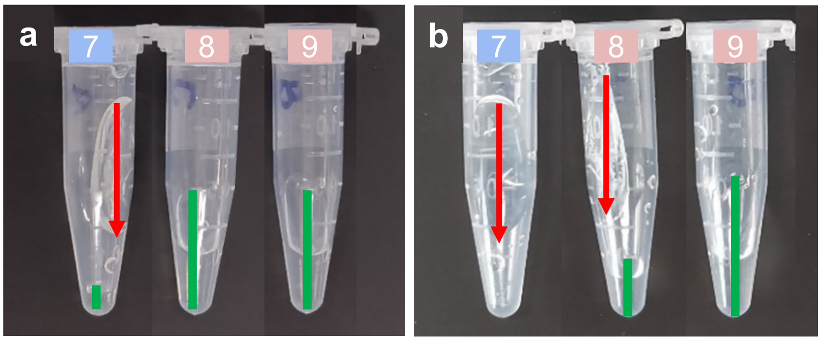

Supplementary Fig. 5 Curing results of different reagent salts in precursor solutions. Photograph of entries 7, 8, 9 in Supplementary Table 4 after 365 nm UV LED irradiating for **a** 4 min and **b** 20 min. Note that cured products and uncured liquid are separately marked by a red arrow and a green line in the figure.

Supplementary Table 5 Curing results of raw materials with different halides.

Entry	Reagent salts	Curing results		
		311 nm light (5 V)	365 nm light (20 W)	405 nm light (3 W)
10	$2MABr+PbBr_2$	Cured/60 s	Cured/20 s	Cured/60 s
11	$2MAI+PbI_2$	Uncured/60 s	Uncured/140 s	Uncured/60 s
12	$2MACl+PbCl_2$	Uncured/60 s	Uncured/140 s	Uncured/60 s

Note: a) all entries above were conducted by blending reagent salts and monomers in the DMSO solvent, where the monomers are equal mole TAIC and TTMP with certain concentrations; b) the numbers before reagent salts are assigned to the ratios. c) 400 μL liquid of each entry was respectively irradiated by 311nm UV LED (5 V), 365 nm UV LED (20 W) and 405 nm UV LED (3 W).

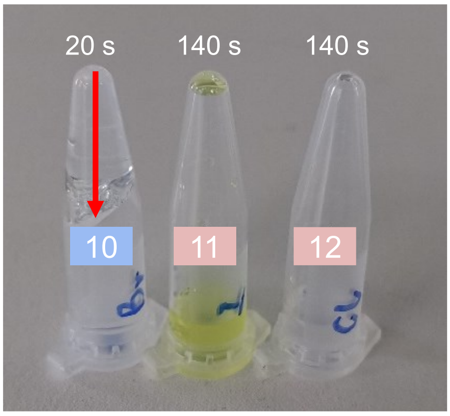

Supplementary Fig. 6 Curing results of raw materials with different halides. Photograph of entries 10, 11, 12 in Supplementary Table 5 after 365 nm UV LED irradiating for 20 s, 140 s,140 s respectively. Note that cured products are marked by a red arrow.

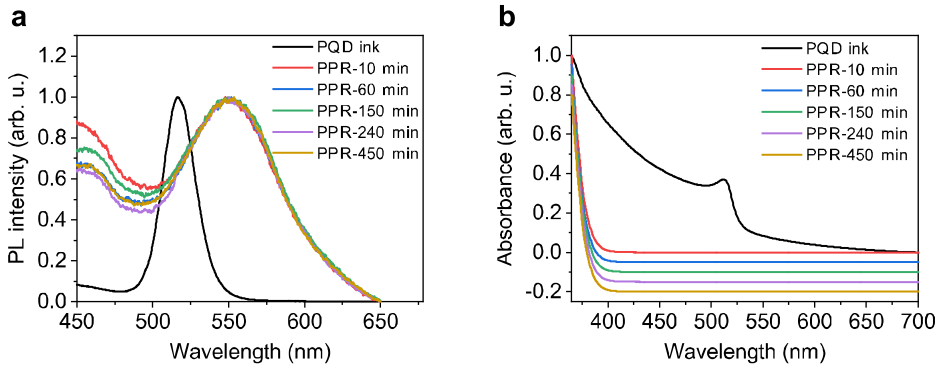

Supplementary Fig. 7 Spectral studies for the PPR evolution with time. **a** PL and **b** UV-Vis spectra of the PPR with increased stirring time and the PQD ink. The PPR was prepared based on the green PPR in the Methods. The PQD ink was prepared by blending the same monomers as PPR and PQDs which were synthesized by the hot injection methods.[1] Source data are provided as a Source Data file.

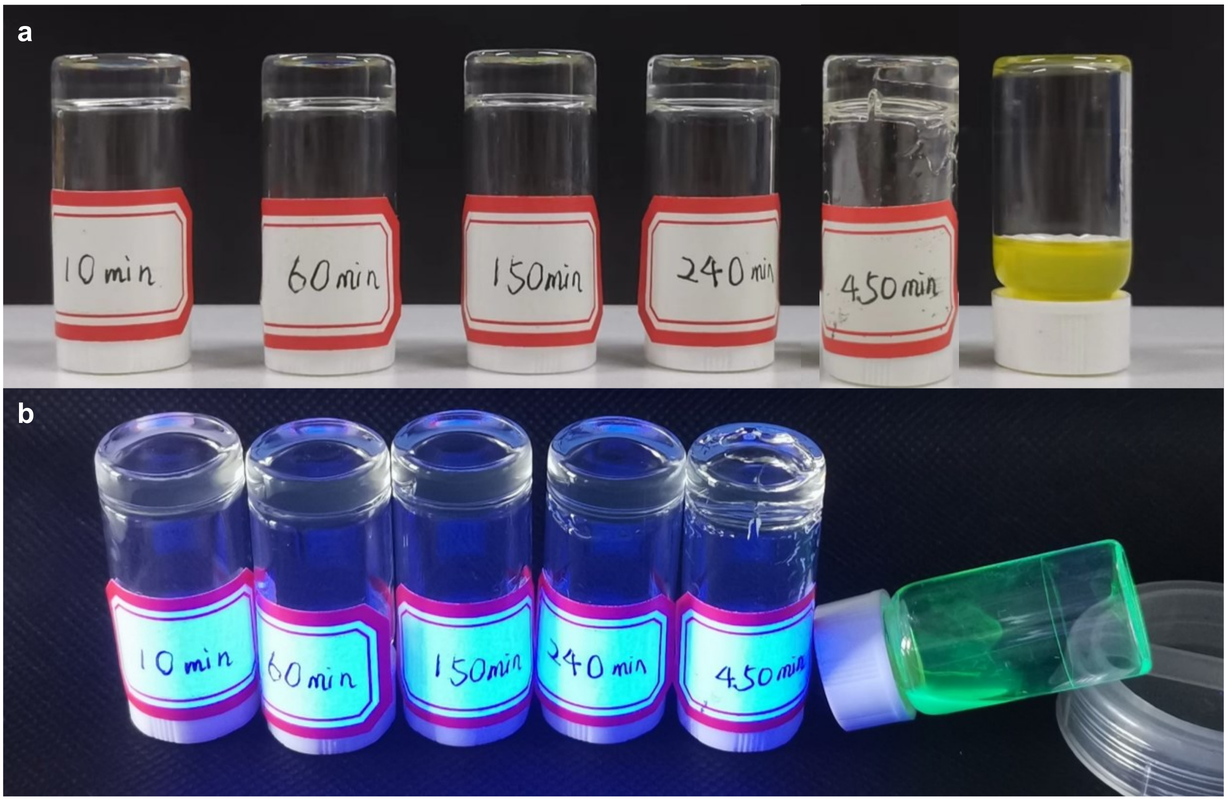

Supplementary Fig. 8 Photographs of curing results of the PPR with varied stirring time and PQD ink under **a** sunlight and **b** UV light. 600 μL liquid samples were irradiated by a 365 nm UV LED (10 W) for 2 min (left five, PPR) and 4 min UV irradiation (the rightest one, PQD ink). The PPR was prepared based on the green PPR in the Methods. The PQD ink was prepared by blending the same monomers as PPR and PQDs which were synthesized by the hot injection methods.[1]

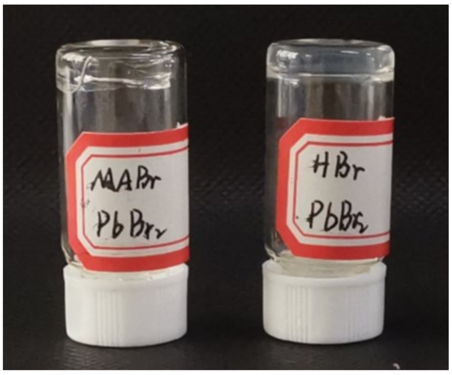

Supplementary Fig. 9 Curing results of different bromide sources in inks: sample (the left one) and sample (the right one) each prepared by adding 0.2 mmol MABr and HBr in 1 mL $PbBr_2$ solution of 0.15 M in DMSO and then mixing with equal amounts of monomers, 550 μL of which were irradiated by a 365 nm UV light (20 W) from the same distance for 30 s.

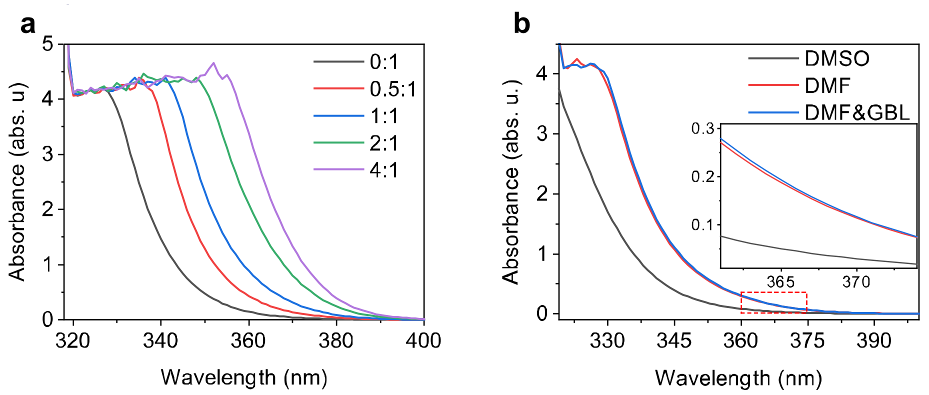

Supplementary Fig. 10 UV-Vis spectra. a PPRs with different ratios of MABr versus $PbBr_2$. All solutions were prepared based on the mixture of 300 μL monomer solution (mole ratio of TAIC to TTMP = 1:1) and 360 μL precursor solutions with a fixed $PbBr_2$ concentration of 0.15 M and altering MABr ratios in DMF and DMSO (v/v = 4:1). **b** PPRs with different solvents. All solutions were prepared on the mixture of 300 μL monomer solution (mole ratio of TAIC to TTMP = 1:1) and 360 μL precursor solutions with a fixed $PbBr_2$ concentration of 0.15 M and a fixed mole ratio of MABr to $PbBr_2$ (2:1), while the solvent was changed from DMSO to DMF to the mixture of DMF and GBL (v/v = 2:3). Source data are provided as a Source Data file.

Supplementary Table 6 pH of different solutions.

Entry	Monomers	Reagent salts	pH
13	2 TAIC	2 MABr + 1 $PbBr_2$	≧5.0
14	2 TTMP	2 MABr + 1 $PbBr_2$	4.0
15	1 TTMP + 1 TAIC	2 $PbBr_2$	4.0

Note: a) all entries above were conducted by blending reagent salts and monomers in the DMSO solvent; b) the numbers before reagent salts and monomers are assigned to the ratios.

Supplementary Table 7 Curing results without and with OA as an additive.

Entry	Additive	Curing results under 365 nm light (~10 mW cm^{-2})
16	None	Cured/6 min
17	OA (2.1 v%)	Cured/10 min

Note: a) all entries above were conducted by adding additives in the $PbBr_2$ and MABr (mole ratio 1:1) solution with a concentration of 0.1 M in DMSO, and mixing them with monomer solution (mole ratio of TAIC to TTMP = 1:1) at a volume ratio of 1.2:1; b) 300 μL liquid of each entry was irradiated under a 365 nm light (~10 mW cm^{-2}).

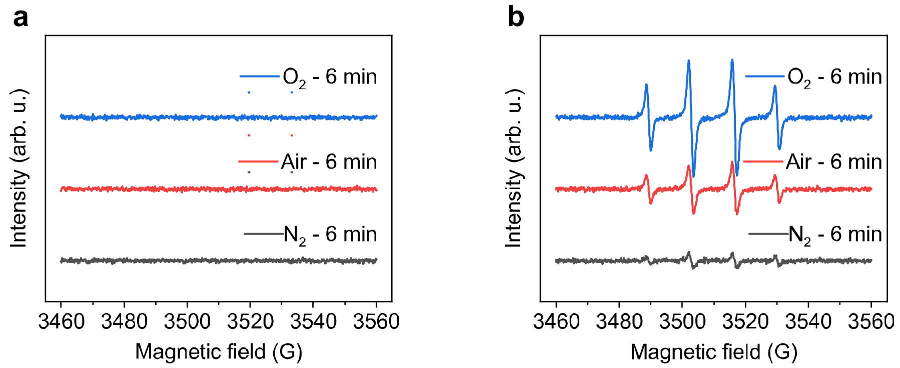

Supplementary Fig 11 EPR spectra for exploration of the effect of O_2. EPR spectra of perovskite precursor solution with TTMP were collected after **a** 0 min and **b** 6 min UV irradiation in the atmosphere of N_2, air and O_2. All solutions were prepared by blending green perovskite precursor solution with TTMP. The testing tubes were each inflated N_2, Air and O_2 for 10 min. 365 nm UV was used to in situ irradiate testing solutions. Source data are provided as a Source Data file.

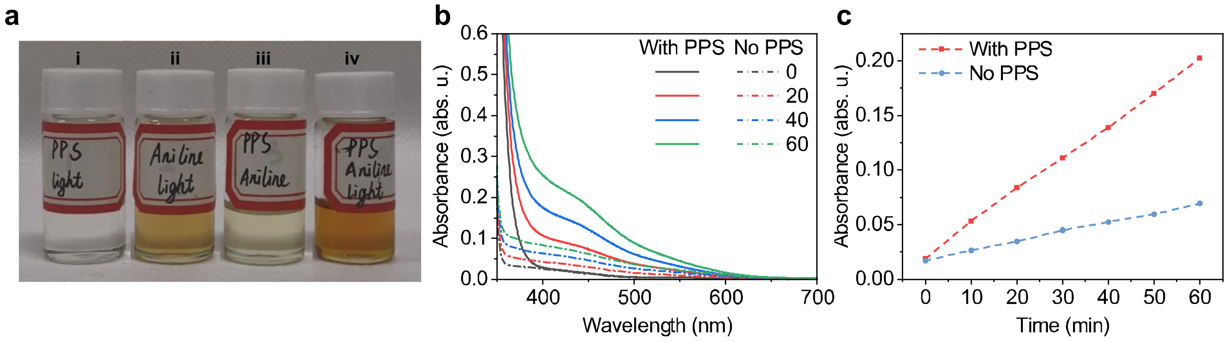

Supplementary Fig. 12 Exploration of the photocatalytic effect of lead bromide complexes on aniline. a Photograph of (i) perovskite precursor solution (PPS) under UV light illumination for 60 min; (ii) solution of aniline under UV light illumination for 60 min; (iii) PPS and aniline under sunlight for 60 min; (iv) PPS and aniline under UV light illumination for 60 min. **b** UV-vis absorption spectra of aniline solution (dot-dash line) and the mixture of PPS and aniline (solid line) in (**a**) under UV light for different reaction time. **c** Time-dependent absorbance profile at 435 nm extracted from (**b**) for tracking polyaniline formation in the presence and absence of PPS. The PPS was prepared by dissolving iso-stoichiometric CsBr and $PbBr_2$ with 0.05 M in DMSO; the aniline solution was prepared by blending aniline with DMSO at a volume ratio of 3:7; solutions in (ii) and (iv) were prepared by blending aniline and PPS at a volume ratio of 3:7. The 365 nm UV LED (~150 mW cm^{-2}) was adopted to illuminate samples at the same time. Source data are provided as a Source Data file.

Supplementary Discussion 1 The photocatalytic effect of lead bromide complexes on aniline

Supplementary Fig. 12a compares the samples with perovskite precursor solution (PPS) and aniline irradiated by UV light for 60 min (iv), and those without aniline (i), PPS (ii), or light (iii), separately. It is clear that the largest change of color, from light yellow to dark brown, was illustrated in the one with both perovskite precursors and aniline under UV irradiation, implying the acceleration of the reaction by applying lead bromide complexes and UV light. Furthermore, the UV spectra and the line graph (Supplementary Fig. 12b, c) also confirmed the acceleration of the photocatalytic effect by lead bromide complexes. This phenomenon can be explained that the lead bromide complexes can deliver holes to the amido of aniline once exposed to UV light. The photocatalytic capacity of lead bromide complexes on aniline enables us to believe that the lead bromide complexes can work on other rich radical reactions.

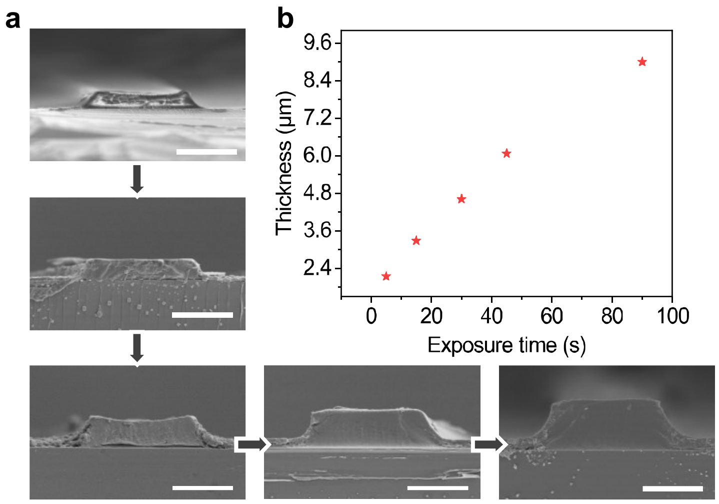

Supplementary Fig. 13 Exploration of the thickness of patterns with respect to UV exposure time. a The cross-section SEM image of stripe patterns under different exposure time (5 s, 15 s, 30 s, 45 s, and 90 s), the scale bar in all micrographs is 10 μm. **b** The thickness of stripes versus corresponding exposure time. All patterned films with a 40 μm period were prepared on the VTMS modified glasses from a blue PPR described in the Methods and processed following the procedure introduced in the Methods with a 365 nm UV LED (~180 mW cm^{-2}). Source data are provided as a Source Data file.

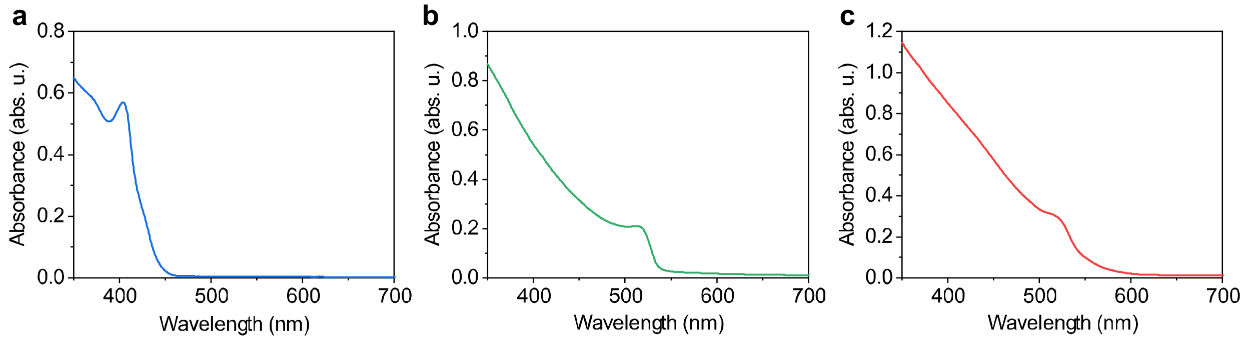

Supplementary Fig. 14 Absorption spectra of the PQD-polymer film. a Blue PQD-polymer film. **b** Green PQD-polymer film. **c** Red PQD-polymer film. All films were prepared on the VTMS modified glasses from blue, green, red PPR described in the Methods and processed follow the procedure introduced in the Methods with a 365 nm UV LED (~180 mW cm^{-2}). Source data are provided as a Source Data file.

Supplementary Table 8 PL intensity distribution for 196 pixels of 20 um blue PQD-polymer dots.

PL intensity range	Count			Average count	SD
	Data set 1	Data set 2	Data set 3		
1800 – 2000	22	10	22	18	6.9
2000 – 2200	132	133	140	135	3
2200 – 2400	39	49	32	40	6.1
2400 – 2600	3	4	2	3	1

Note: all data were counted by ImageJ software based on the patterned aways of blue PQD-polymer dots prepared according to the Methods. Errors were determined by standard deviantion (SD) and were illustrated in Fig. 4e as error bars. The origin PL intensity of 196 pixels were presented in Soure Data file.

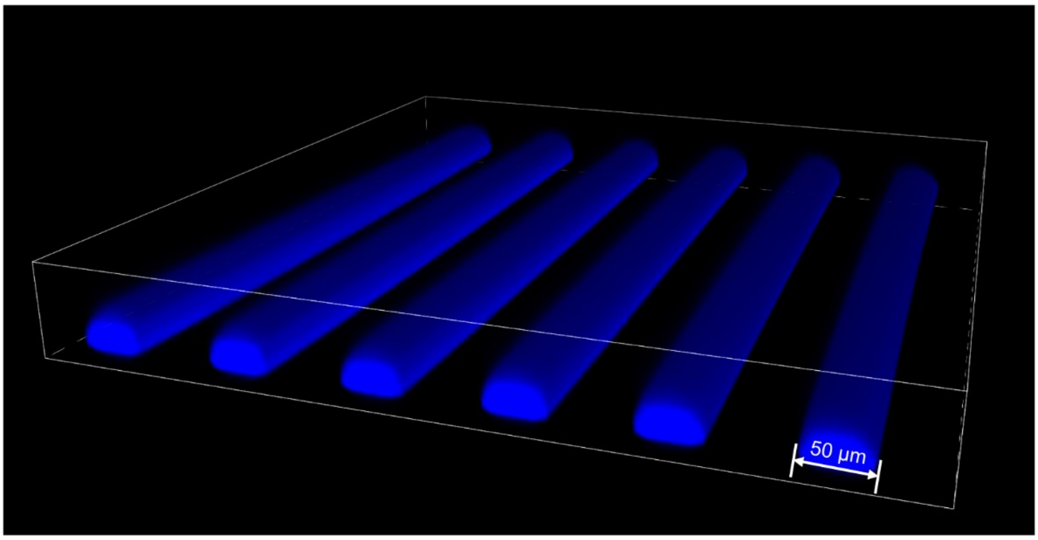

Supplementary Fig. 15 Laser scanning confocal 3D microscope image of the blue PQD-polymer film with stripes of 100 μm period on the VTMS modified glass, which was prepared based on the direct in-situ photolithography method with the blue PPR described in the Methods.

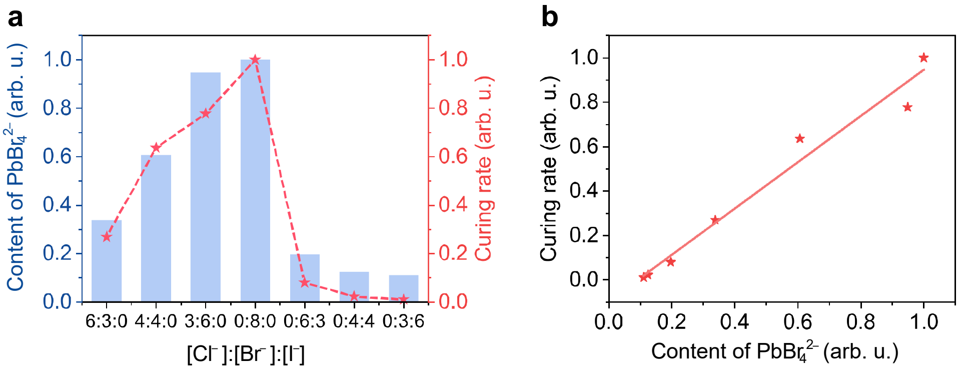

Supplementary Fig. 16 The effect of replacing Br^- with Cl^- or I^- on the content of $PbBr_4^{2-}$ in solutions and the curing rate of related PPRs. a The content of $PbBr_4^{2-}$ in precursor solutions (column chart) and curing rate of corresponding PPRs (line chart) versus different ratios of [Cl^-]:[Br^-]:[I^-]. **b** The curing rate versus the content of $PbBr_4^{2-}$ extracted from (**a**), fitting with a straight line. The content of $PbBr_4^{2-}$ in precursor solutions was determined by the PL intensity, and the precursor solutions were prepared by dissolving MAX and PbX_2 (n/n = 2:1) in DMSO and DMF (v/v =1:1) at the fixed lead concentration of 0.1 M. The PPRs for the curing experiment were prepared by blending equal volume of precursor solutions and the monomer solution with TTMP and TAIC (n/n = 1:1). Then they were irradiated under 365 nm UV light to obtain the curing time. The curing rate was calculated by inverting the curing time. Source data are provided as a Source Data file.

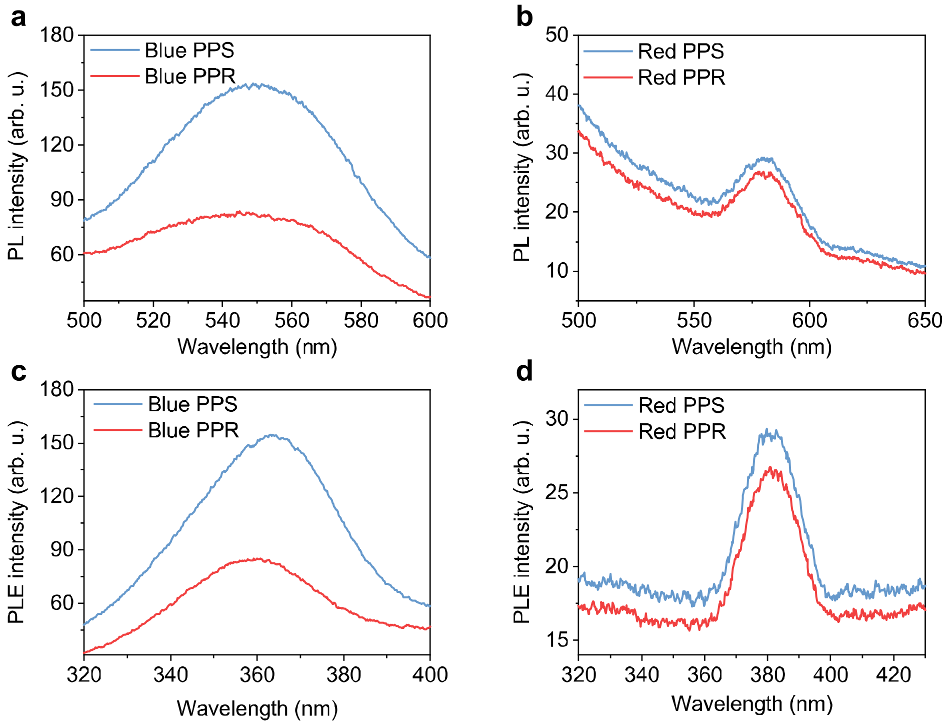

Supplementary Fig. 17 Spectra of blue and red perovskite precursor solutions (PPSs) and PPRs. PL spectra of **a** the blue PPS and PPR, and **b** the red PPS and PPR; PLE spectra of **c** the blue PPS and PPR, and **d** the red PPS and PPR. All PPRs were prepared based on the constitution and conditions of the blue and red PPRs in the Methods, and the corresponding PPSs were just prepared based on the constitutions of the PPRs without any monomers in the same conditions. Source data are provided as a Source Data file.

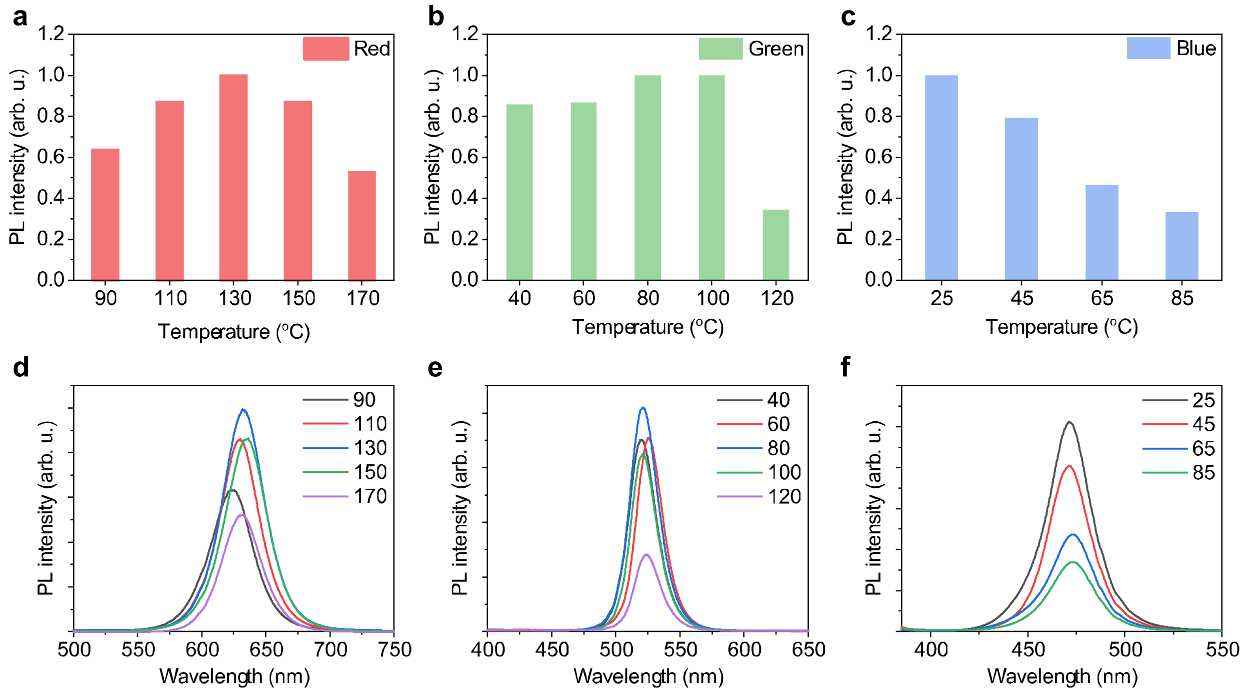

Supplementary Fig. 18 Influence of annealing temperature on PL. PL intensity of **a** $MA_xCs_{1-x}PbI_yBr_{3-y}$-polymer films, **b** $MAPbBr_3$-polymer films and **c** $MAPbBr_xCl_{3-x}$-polymer films. PL spectra of **d** $MA_xCs_{1-x}PbI_yBr_{3-y}$-polymer films, **e** $MAPbBr_3$-polymer films and **f** $MAPbBr_xCl_{3-x}$-polymer films. All films were fabricated via the direct in-situ photolithography method with the red, green, and blue PPRs depicted in the Methods with the exception of altering annealing temperatures. Source data are provided as a Source Data file.

Supplementary Discussion 2 Influence of annealing temperature on PL

To explore the relationship between the formation of PQDs and the annealing temperature, PQD films at different annealing temperatures were fabricated. For red perovskite, an intermediate phase tends to form after anti-solvent dripping due to interaction between Lewis base DMSO and/or iodide (I^-) and Lewis acid PbI_2.[2, 3] The PL intensity increased with increasing annealing temperature from 90°C to 130°C (Supplementary Fig. 18a), which is possibly related to the increasing conversion of the intermediate phase to the pure phase and the growth of nuclei into the larger nanocrystals. With further elevated temperature, the PL intensity presented a downward trend, which can be attributed to the thermal decomposition. Therefore, 130°C was chosen as the annealing temperature to fabricate red PQDs. By comparison, green and blue PQDs with lower solubility and formation enthalpy can be formed directly from the disordered solvate phase during solution processing,[4] indicating lower temperature is needed. For green PQDs, the increasing PL intensity before 80°C can also be ascribed to the removal of remained solvents and the growth of PQDs. After that, a drop of the PL intensity was observed, possibly due to the aggregation of PQDs and the partial sublimation of MABr thus inducing the degradation of the PQDs. We fabricated the blue PQDs with mixed-halide of

chloride/bromide. Since blue PQDs have the poorest solubility[5] and negative formation enthalpy,[6] they can crystalize facilely even at room temperature. In the mixed-halide perovskite, Cl^{-} and Br^{-} possess different migration rates due to the varied ion radius and binding energy, resulting in phase separation.[7] Heat can accelerate the phase separation, consistent with the decline in the PL intensity with increasing temperature in blue PQDs (Supplementary Fig. 18c).

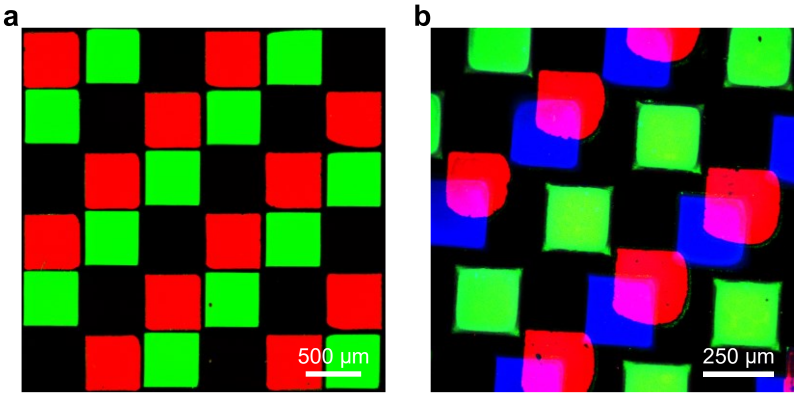

Supplementary Fig. 19 Fluorescence images of multiple color PQD patterns. **a** Red and green double color patterned film with squares of 500 μm. **b** Red, green and blue three color patterned film with squares of 250 μm. An intermediate layer of 300 nm thickness SiO_2 was deposited before patterning another emitting layer, which did not affect the optical properties of the final patterns.

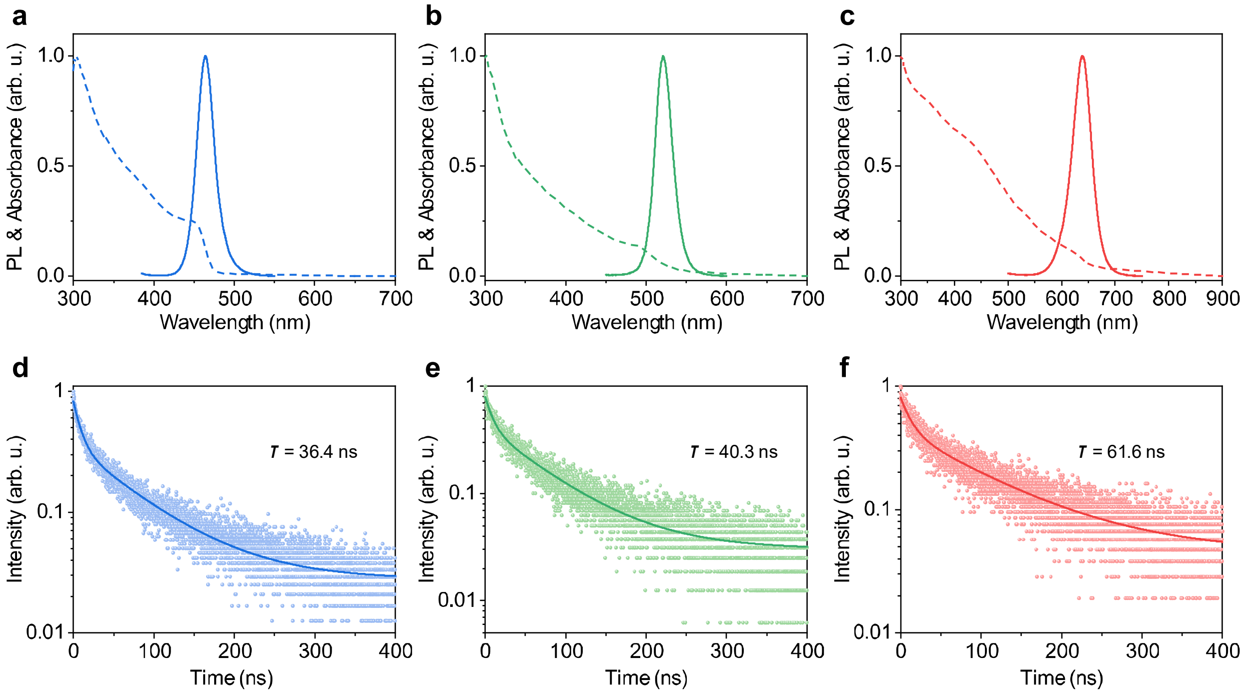

Supplementary Fig. 20 Spectra of patterned PQD films via direct in-situ photolithography. PL and UV-Vis spectra of **a** blue, **b** green, **c** red patterned films. Time-resolved PL spectra of **d** blue, **e** green, **f** red patterned films. All films were patterned via the direct in-situ photolithography method with the blue, green and red PPRs according to the Methods. Source data are provided as a Source Data file.

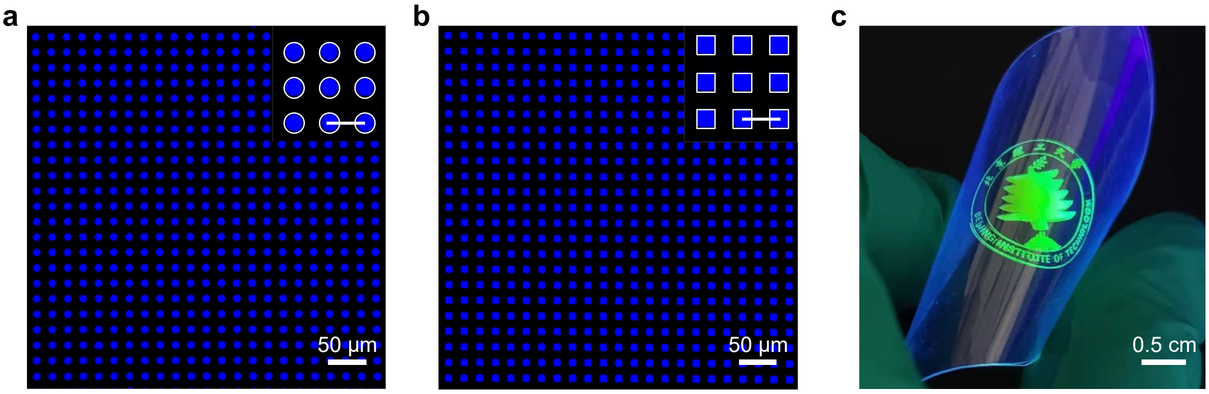

Supplementary Fig. 21 Fluorescence image of PQD-polymer films. Fluorescence optical microscope image of **a** blue circle patterns with a diameter of 10 µm, **b** blue square patterns with a size of 10 µm, the scale bars in the (**a**), (**b**) inset images are 20 µm. **c** Patterns on the flexible PET under UV light.

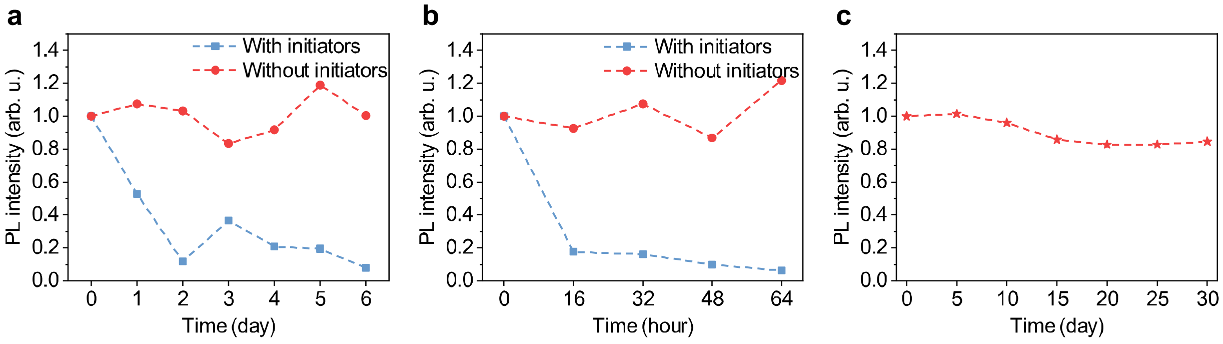

Supplementary Fig. 22 Stability test of $MAPbBr_3$ PQD-polymer films to UV light exposure, heat and atmosphere. **a** Remnant PL intensity for films with and without initiators versus time under 365 nm UV light irradiation (~150 mW cm^{-2}). **b** Remnant PL intensity versus heating time at 60°C. **c** Remnant PL intensity for the film without initiators versus storing time in atmosphere with average humidity of 54%. All films were prepared based on the direct in-situ photolithography method with the green PPRs described in the Methods, while the films labeled "with initiators" in the graphs were deliberately introduced 2 w% initiators. Source data are provided as a Source Data file.

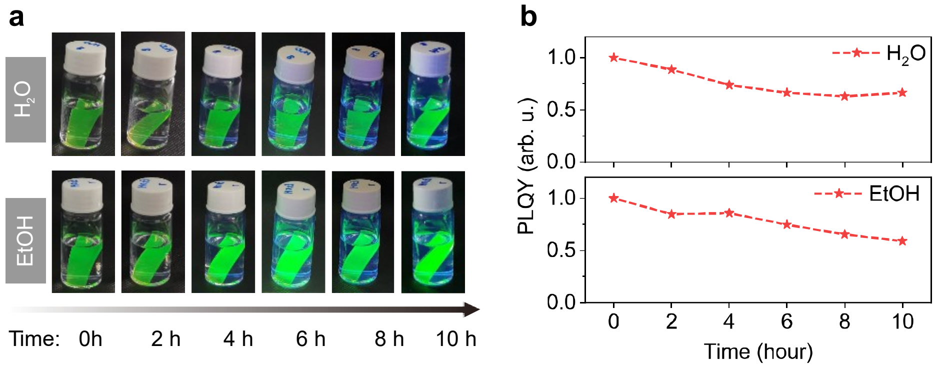

Supplementary Fig. 23 Stability test for MAPbBr3-polymer films against deterioration of solvents. a Fully exposed films fabricated via the direct in-situ photolithography method were completely soaked at rest in the water and ethanol (EtOH) at room temperature. **b** Remnant PL QY recorded from (**a**). Source data are provided as a Source Data file.

Supplementary References

1. Protesescu, L. *et al.* Nanocrystals of cesium lead halide perovskites ($CsPbX_3$, X = Cl, Br, and I): novel optoelectronic materials showing bright emission with wide color gamut. *Nano Lett.* **15**, 3692-3696 (2015).
2. Ahn, N.-. Highly reproducible perovskite solar cells with average efficiency of 18.3% and best efficiency of 19.7% fabricated via lewis base adduct of lead(II) iodide. *J. Am. Chem. Soc.* **137**, 8696-8699 (2015).
3. Jeon, N. J. *et al.* Solvent engineering for high-performance inorganic–organic hybrid perovskite solar cells. *Nat. Mater.* **13**, 897-903 (2014).
4. Zhang, S. *et al*. Wide-band-gap mixed-halide 3D perovskites: electronic structure and halide segregation investigation. *ACS Appl. Electron. Mater.* **3**, 2277-2285 (2021).
5. Stevenson, J. *et al*. Mayer bond order as a metric of complexation effectiveness in lead halide perovskite solutions. *Chem. Mater.* **29**, 2435-2444 (2016).
6. Nagabhushana, G. P., Shivaramaiah, R. & Navrotsky, A. Direct calorimetric verification of thermodynamic instability of lead halide hybrid perovskites. *Proc. Natl. Acad. Sci. U. S. A.* **113**, 7717-7721 (2016).
7. Zhang, F. *et al*. Chlorine distribution management for spectrally stable and efficient perovskite blue light-emitting diodes. *Nano Energy* **79**, 105486 (2021).
