# Supplementary material for: Direct in situ photolithography of perovskite quantum dots based on photocatalysis of lead bromide complexes
Source: Nat Commun. 2022 Nov 7;13:6713. doi: 10.1038/s41467-022-34453-9 (PMC9640639; doi:10.1038/s41467-022-34453-9)
Supplement: Supplementary file 3 — Reporting Summary [file 41467_2022_34453_MOESM3_ESM.pdf]

nature portfolio

Corresponding author(s): Gaoling Yang

Last updated by author(s): Oct 4, 2022

Reporting Summary

Nature Portfolio wishes to improve the reproducibility of the work that we publish. This form provides structure for consistency and transparency in reporting. For further information on Nature Portfolio policies, see our Editorial Policies and the Editorial Policy Checklist.

Statistics

For all statistical analyses, confirm that the following items are present in the figure legend, table legend, main text, or Methods section.

n/a	Confirmed	
☐	☒	The exact sample size (*n*) for each experimental group/condition, given as a discrete number and unit of measurement
☐	☒	A statement on whether measurements were taken from distinct samples or whether the same sample was measured repeatedly
☐	☒	The statistical test(s) used AND whether they are one- or two-sided<br>*Only common tests should be described solely by name; describe more complex techniques in the Methods section.*
☐	☒	A description of all covariates tested
☐	☒	A description of any assumptions or corrections, such as tests of normality and adjustment for multiple comparisons
☐	☒	A full description of the statistical parameters including central tendency (e.g. means) or other basic estimates (e.g. regression coefficient) AND variation (e.g. standard deviation) or associated estimates of uncertainty (e.g. confidence intervals)
☒	☐	For null hypothesis testing, the test statistic (e.g. *F*, *t*, *r*) with confidence intervals, effect sizes, degrees of freedom and *P* value noted<br>*Give P values as exact values whenever suitable.*
☒	☐	For Bayesian analysis, information on the choice of priors and Markov chain Monte Carlo settings
☒	☐	For hierarchical and complex designs, identification of the appropriate level for tests and full reporting of outcomes
☒	☐	Estimates of effect sizes (e.g. Cohen's *d*, Pearson's *r*), indicating how they were calculated

Our web collection on statistics for biologists contains articles on many of the points above.

Software and code

Policy information about availability of computer code

Data collection	No software was used
Data analysis	Origin 2018 64bit

For manuscripts utilizing custom algorithms or software that are central to the research but not yet described in published literature, software must be made available to editors and reviewers. We strongly encourage code deposition in a community repository (e.g. GitHub). See the Nature Portfolio guidelines for submitting code & software for further information.

Data

Policy information about availability of data

All manuscripts must include a data availability statement. This statement should provide the following information, where applicable:

- Accession codes, unique identifiers, or web links for publicly available datasets
- A description of any restrictions on data availability
- For clinical datasets or third party data, please ensure that the statement adheres to our policy

The data generated in this study are provided in the Source Data file. Source data are provided with this paper.

Human research participants

Policy information about studies involving human research participants and Sex and Gender in Research.

Reporting on sex and gender	This information has not been collected.
Population characteristics	See above
Recruitment	See above
Ethics oversight	See above

Note that full information on the approval of the study protocol must also be provided in the manuscript.

Field-specific reporting

Please select the one below that is the best fit for your research. If you are not sure, read the appropriate sections before making your selection.

☐ Life sciences ☐ Behavioural & social sciences ☒ Ecological, evolutionary & environmental sciences

For a reference copy of the document with all sections, see nature.com/documents/nr-reporting-summary-flat.pdf

Ecological, evolutionary & environmental sciences study design

All studies must disclose on these points even when the disclosure is negative.

Study description	Herein, we report a direct in-situ photolithography technique to pattern PQDs based on the photopolymerization catalyzed by lead bromide complexes. By combining direct photolithography with in situ fabrication of PQDs, this method allows to directly photolithograph perovskite precursors, avoiding the complicated lift-off processes and the destruction of PQDs by solvents or high-energy UV light, as PQDs are produced after lithography exposure. We further demonstrate that the thiol-ene free-radical photopolymerization is catalyzed by lead bromide complexes in the perovskite precursor solution, while no external initiators or catalysts are needed. Using direct in-situ photolithography, PQD patterns with high resolution up to 2450 pixels per inch (PPI), excellent fluorescence uniformity, and good stability, are successfully demonstrated.
Research sample	We mixed perovskite precursors with monomers in the solvents to obtain the perovskite precursor resist. Perovskite quantum dots were fabricated in situ in the polymer.
Sampling strategy	This is not relevant to the present study.
Data collection	Data were collected by Pingping Zhang.
Timing and spatial scale	Data were collected in the laboratory
Data exclusions	No data were excluded in the analyses.
Reproducibility	The reproducibility of the samples has been done at least three times independently.
Randomization	This is not relevant to the present study.
Blinding	This is not relevant to the present study.

Did the study involve field work? ☐ Yes ☒ No

Reporting for specific materials, systems and methods

We require information from authors about some types of materials, experimental systems and methods used in many studies. Here, indicate whether each material, system or method listed is relevant to your study. If you are not sure if a list item applies to your research, read the appropriate section before selecting a response.

Materials & experimental systems

n/a	Involved in the study
☒	☐ Antibodies
☒	☐ Eukaryotic cell lines
☒	☐ Palaeontology and archaeology
☒	☐ Animals and other organisms
☒	☐ Clinical data
☒	☐ Dual use research of concern

Methods

n/a	Involved in the study
☒	☐ ChIP-seq
☒	☐ Flow cytometry
☒	☐ MRI-based neuroimaging
